# The Genetic Basis of Natural Variation in *Drosophila melanogaster* Immune Defense against *Enterococcus faecalis*

**DOI:** 10.3390/genes11020234

**Published:** 2020-02-22

**Authors:** Joanne R Chapman, Maureen A Dowell, Rosanna Chan, Robert L Unckless

**Affiliations:** 1 Department of Molecular Biosciences, University of Kansas, Lawrence, Kansas, KS 66045, USA; maureen_dowell@brown.edu (M.A.D.); rosannachan01@gmail.com (R.C.); unckless@ku.edu (R.L.U.); 2 Department of Molecular Biology, Cell Biology & Biochemistry, Brown University, Providence, Rhode, RI 02912, USA

**Keywords:** genome-wide association study (GWAS), innate immunity, immune response, systemic infection, Toll pathway, gram-positive bacteria

## Abstract

Dissecting the genetic basis of natural variation in disease response in hosts provides insights into the coevolutionary dynamics of host-pathogen interactions. Here, a genome-wide association study of *Drosophila melanogaster* survival after infection with the Gram-positive entomopathogenic bacterium *Enterococcus faecalis* is reported. There was considerable variation in defense against *E. faecalis* infection among inbred lines of the *Drosophila* Genetics Reference Panel. We identified single nucleotide polymorphisms associated with six genes with a significant (*p* < 10^−08^, corresponding to a false discovery rate of 2.4%) association with survival, none of which were canonical immune genes. To validate the role of these genes in immune defense, their expression was knocked-down using RNAi and survival of infected hosts was followed, which confirmed a role for the genes *krishah* and *S6k* in immune defense. We further identified a putative role for the Bomanin gene *BomBc1* (also known as *IM23*), in *E. faecalis* infection response. This study adds to the growing set of association studies for infection in *Drosophila melanogaster* and suggests that the genetic causes of variation in immune defense differ for different pathogens.

## 1. Introduction

Pathogenic microorganisms, those that cause increased morbidity and/or mortality in infected individuals, can pose serious threats to the fitness of infected individual hosts and the long-term survival of host populations, and even species [1,2]. In response to the challenges posed by pathogens, most hosts have developed a complex series of defense mechanisms, collectively known as the immune system. Yet, mounting an immune response to pathogens is costly in terms of both energy expenditure and resource allocation [3,4]. For this reason, selection should act on hosts to tolerate or resist pathogens based on the relative costs of harboring or clearing an infection. Furthermore, within host populations there tends to be genetic variation in immune defense, such that individuals vary in their response to specific pathogens. This variation in immune defense is not simply restricted to canonical immunity genes, but also to those genes involved in life-history, behavioral and physiological traits that influence the outcome of host-pathogen interactions [5]. Continual host-pathogen coevolution likely promotes maintenance of immune diversity in populations [6,7]. Understanding the genetic underpinnings of natural variation for immunity within a population or species therefore provides insights into the specific genes and immune pathways involved in the host immune response to a particular pathogen.

Genome-wide association studies (GWAS) test for statistical associations between genotype and phenotype at each variable site in the genome, and theoretically have the power to detect alleles with only modest effects on phenotype [8]. One perceived benefit of GWAS is that it allows for unbiased detection of unknown and unexpected genotypic associations because the whole genome is interrogated simultaneously. While this method has been used to examine the genetic basis of immunity in *Drosophila melanogaster* previously e.g., [9,10,11,12,13], no study to date has used GWAS to examine *Drosophila* defense against *Enterococcus faecalis*. 

*E. faecalis* is a Gram-positive bacterium with lysine-type peptidoglycan that readily infects insect and mammalian hosts [14], where it lowers individual survival [14,15]. *E. faecalis* occurs naturally in *Drosophila* populations, most likely at low prevalence [16,17]. Importantly, *E. faecalis* has been isolated from the hemolymph of wild *D. melanogaster*, and challenge with this bacterium at a relatively low dose causes intermediate levels of mortality in laboratory reared flies [17,18]. Thus, *E. faecalis* is not solely a gut commensal of *D. melanogaster* but rather can cause systemic pathogenic infections. As such, it is likely that there is selection for resistance and/or tolerance in *D. melanogaster*. Indeed, Toll-pathway induced antimicrobial peptides are upregulated in response to *E. faecalis* infection and play a key role in controlling the infection [19,20,21]. Recently, a new group of immune peptides, the Bomanins, were shown to play an important role in controlling Gram-positive infections such as *E. faecalis* in *D. melanogaster* [15,22,23]. 

Here, we use a genome-wide association study to identify genetic variation involved in *D. melanogaster* immune defense against infection with *E. faecalis*. Subsequently, expression of putative response genes was knocked down in vivo using RNAi and survival after infection was monitored. This allowed us to confirm the role of two genes involved in *D. melanogaster* survival after *E. faecalis* infection that have not previously been recognized as playing a role in immunity to this pathogen. In addition, we dissected the role that Bomanins play in regulating response to *E. faecalis* infection and showed *BomBc1* may play a specific role in enhancing *Drosophila* survival when infected with this pathogen.

## 2. Materials and Methods 

### 2.1. Drosophila and Bacterial Stocks

The *Drosophila* Genetic Reference Panel (DGRP) population was used in this study. The DGRP is a North American (Raleigh, NC, USA) set of inbred lines that was developed for analysis of quantitative traits [24]. Full genome sequences are available for every line (*n* = 205) [25]. Flies were housed at 22 °C on a 12-h day/night cycle in vials containing standard *Drosophila* cornmeal-molasses-yeast food.

The *E. faecalis* strain used in this study was originally isolated from the hemolymph of wild-caught *D. melanogaster* [17]. One week before the start of the experiment, the bacterium was revived from a glycerol stock maintained at −80 °C by streaking out on a Lysogeny Agar plate (LA, Difco, Detroit, MI, USA) and incubating the plate at 37 °C overnight. This plate was then stored at 4 °C. The evening before infections were to be performed, a single colony was aseptically picked from the LA plate and placed in 2 mL of sterile Lysogeny broth (LB, Difco, Detroit, MI, USA) at 37 °C with shaking (250 rpm) overnight. The next day, the bacterial suspension was spun down (1 min at 13,000 rpm) and resuspended in LB to a final OD_600_ of 1.5 ± 0.02, which represents a final bacterial concentration of ~1.3 × 10^9^ CFU/mL. 

### 2.2. Genome-Wide Association Study

All but three DGRP lines (DGRP_21, DGRP_49, DGRP_325) were included in this study, resulting in an initial dataset of 202 DGRP lines in the GWAS analysis. Four infectors (JRC, MAD, RC, RLU) aimed to each infect at least 10 individuals per line. Adult males were infected 4–7 days post-eclosion, via piercing of the thorax with a Ø 0.1 mm pin that had been dipped in the bacterial suspension described above. We limited all our infection studies to males because female flies can exhibit different immune responses depending on mating status and reproductive investment [26,27,28]. We used 4–7-day-old adult flies because the fat body (the main immune tissue in *Drosophila*) is not fully mature and fixed for the first three days of the adult stage [29] and because flies undergo immunosenescence as they age [30]. Four to seven days, therefore, represents a period of life in which expression of immune genes should be optimal. Other flies were pricked with sterile LB to serve as uninfected controls (mean ± s.d. = 15.95 ± 5.58 per line). After treatment, 4–10 males from a given line (mean ± s.d. = 9.93 ± 0.45) were placed in a vial containing cornmeal-molasses-yeast food, with each vial containing flies subjected to a single treatment by a single infector. Vials were then stored in an incubator at 22 °C on a 12 h day/night cycle. The number of surviving individuals was recorded five days after treatment, which is a reasonable proxy of infection outcome because numerous *Drosophila* infection studies have shown that flies tend to either die of infection within 48 h or survive beyond seven days, e.g., [22,31,32]. We found that DGRP line DGRP_321 was an extreme outlier in that most individuals died shortly after infection. This line has also been previously identified as having abnormally lowered immune functioning [13]. As such, we excluded DGRP_321 and conducted the GWAS analysis on the remaining 201 lines.

To determine whether *E. faecalis* survival is associated with a specific genetic variant in the DGRP, a generalized linear mixed model with a binomial distribution and a logit link was fitted to the survival data using Equation (1):

Proportion alive ~ Date + Infector + *Wolbachia* status + (1| Line)/variant, (1)

where (1|Line)/variant refers to DGRP fly line nested within genetic variant (random effect), variant is the specific variant (A, C, T, or G for SNPs, longer sequence strings for insertions and deletions) at a given genomic position (fixed effect), Date is the day infections were conducted (fixed effect), Infector is the identity of the person performing infections for that vial (fixed effect) and *Wolbachia* status refers to whether the line is systemically infected with the intracellular bacterium *Wolbachia *pipientis** (fixed effect). This test was performed for every variable position in the genome that met the following criteria: A) the site was biallelic amongst the phenotyped lines and B) the minor allele count was greater than five. This led to a total of *n* = 2,383,736 independent tests. 

### 2.3. RNAi Knock-Down of Candidate Genes

An initial list of variants putatively associated with *E. faecalis* survival in the DGRP was generated by applying a *p*-value cut-off of *p* < 10^−7^, corresponding to a false discovery rate of 23.8%. To further winnow the list of variants to validate, we then applied a second, more conservative *p*-value cut-off of *p* < 10^−8^ to reduce the false discovery rate to 2.38% and therefore retain only those variants with a strong association with survival after *E. faecalis* infection. Genes associated with variants significant at the *p* < 10^−8^ level are hereafter termed genes of interest (GOIs). We chose to further dissect the role of a subset of these genes, as well as genes physically close to these candidate genes (which we termed neighbor genes), by knocking down their expression in-vivo using RNAi and following survival after infection with *E. faecalis* in comparison to ‘empty cassette’ controls (see below). The neighbor gene approach was chosen to control for the possibility that knocking down random genes produced a change in immune phenotype. To this end, we used flies created by the Transgenic RNAi Programme (TRiP) [33] which we obtained from the Bloomington *Drosophila* Stock Center. TRiP uses a Gal4-UAS system to drive down expression of the targeted gene when crossed to a driver line. We used two different driver lines, one (Act5C) targets knockdown across the whole body, the other (C564), targets the fat body. We used the fat body driver in cases where whole body knock-down of a gene was lethal, because the fat body is the major immune-related tissue in *Drosophila*, analogous to the mammalian liver [34,35]. To obtain knockdown F1 individuals, RNAi TRiP males were crossed to virgin females from the driver lines Act5C or C564, per gene of interest and ‘empty cassette’ controls (isogenic to the knockdown lines except for the specific hairpin-containing transgene) (Figure S1). F1 offspring from these crosses were collected onto fresh food 1–2 day-post-eclosion for subsequent infection (Figure S1). A balancer chromosome was present in some of the TRiP lines, in these cases we only infected FI progeny with the wild-type phenotype, because these individuals harbor the target RNAi transgene.

### 2.4. *E. faecalis* Infection of RNAi Lines with Knock-Down of Candidate Genes

As with the GWAS analysis, we infected 4–7-day-old males, in this case, F1 offspring from the crosses described above, with *E. faecalis* at a standardized concentration of OD_600_ = 1.5 ± 0.02 and counted the number of survivors after five days. However, for these experiments, only two people infected flies (JRC and MAD). Three types of comparisons were made. First, comparisons were made between lines with identical genetic backgrounds except for the knock-down of one specific gene (target vs ‘empty cassette’ comparisons, where the target is either the GOI or neighbor gene, see below). Second, comparisons were made between knockdowns of the candidate GOI and a neighbor gene, being the closest genomic neighbor gene that was not itself identified in the GWAS (GOI vs neighbor comparisons). Third, lines were infected with either *E. faecalis* or sterile LB (infected vs sterile prick controls, data not shown). In total, we aimed to infect a minimum of 100 males per line (target/empty cassette per GOI/neighbor) with *E. faecalis* and a minimum of 30 males per line (target/empty cassette per GOI/neighbor) with LB (see Table S2), resulting in a minimum of 260 treated flies per experiment. 

To determine whether GOI lines (i.e., those with expression of the candidate gene knocked-down by RNAi) had different survival than control lines without that gene knocked down, a generalized linear model with a binomial distribution and a logit link was fitted to the survival data. Depending on the number of infectors, we used Equation (2) (one infector) or Equation (3) (two infectors):

Proportion alive ~ Line + Date,(2)

Proportion alive ~ Line + Infector/Date,(3)

where Line is the identity of the line (target or control) and Date is the date infections were performed. Infector/Date is date nested within infector. Equation (3) was only required for the analysis of neighbor gene *CG42272*, because both JRC and MAD infected flies for this line (note that only a single person infected on any given date). For all other genes, Equation (2) was fitted because a single person performed all infections. *Wolbachia* status was not required because *Wolbachia* is maternally transmitted and the two driver lines (Act5C and C564) are *Wolbachia* free. To visualize the differences in survival between target RNAi knockdown genes (GOI/neighbor) and empty cassette controls, we fitted a model using Equation (2) or Equation (3) as appropriate, then a second model without the Line effect. We used the residuals from this second model as the response (y) variable and plotted this against the Line effect from the full (Equation (2)/Equation (3)) model as the predictor (x) variable. 

### 2.5. Effects of Knocking Down Specific Bomanin Genes on *E. faecalis* Survival

We additionally investigated the effect of transgenically reducing expression of four Bomanin genes (*BomS1, BomBc1, BomT1* and *BomS4*) by knocking down expression, again using TRiP line males crossed to C564 females and infecting F1 males. Comparisons were made between specific Bom-individuals and control individuals containing the TRiP empty cassette, infected with *E. faecalis* at the standard dose (OD_600_ = 1.5 ± 0.02) or Todd-Hewitt (TH) broth as a sterile control. TH was used in this experiment because it was found that *E. faecalis* grows better in this media. The use of LB or TH as growth medium and sterile prick control had no effect on survival of infected or control flies (data not shown). Five-day survival was recorded. Subsequently, we increased the dose to (OD_600_ = 2.4 ± 0.02) to increase mortality rates, and again recorded survival after five days. All infections were conducted by JRC for this experiment. 

To determine whether knock-down of specific Bomanin genes by RNAi had an effect on fly survival, a generalized linear model with a binomial distribution and a logit link was fitted to the survival data using Equation (2). In this case, Line was the identity of the Bomanin knock-down or control line. 

### 2.6. Statistical Analyses

All statistical analyses were performed using R v 3.4.1 [36]. Generalized models were fitted using the glmer function in the lme4 package [37] and graphics were plotted using ggplot2 [38] and corrplot [39]. 

## 3. Results

### 3.1. Variation in Immunity to *E. faecalis* Infection in the DGRP

**Figure 1:**
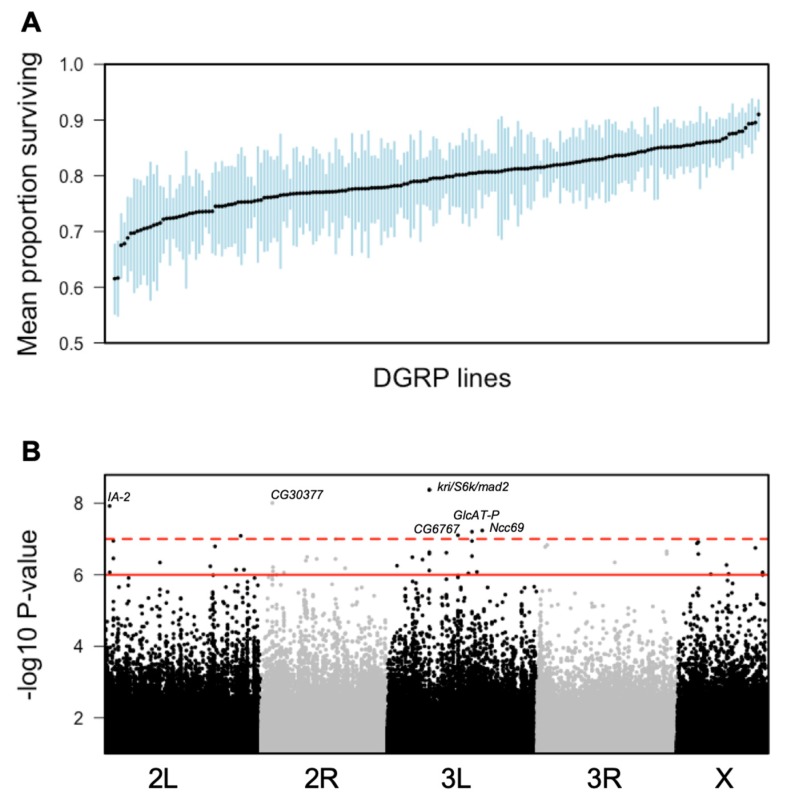
Genome-wide association study (GWAS) analysis using fitted data. (**A**) Distribution of survival rates amongst DGRP lines infected with *E. faecalis* after fitting a model to control for the effects of Date, Infector and *Wolbachia* status. Black line represents fitted average for a model of survival taking into account the fixed effects of infector and date of infection. The plot is sorted by increasing survival, and blue bars indicate standard error of the mean, per line. (**B**) Manhattan plot of genetic variants across the *D. melanogaster* genome and their association with *E. faecalis* survival. *p*-value is plotted as −log_10_ *p*-value. Solid red line indicates *p*-value cut-off of 10^−7^ (which equates to a −log_10_ *p*-value of 6). Points above this line were used for generating list of variants putatively associated with survival after infection (see Table S1). Dashed red line indicates *p*-value cut-off of 10^−8^ (which equates to a −log_10_ *p*-value of 7). Most variants above this line were selected for candidate gene analysis; variants associated with genes are labelled with gene name. Black and grey shading delineates chromosomes.

In total, 27–65 males were infected with *E. faecalis* per line (mean ± s.d. = 46.32 ± 6.02, Figure S2a). There was a more than three-fold difference in raw survivorship across the DGPR lines in their ability to survive infection for five days (Figure S2b). The five lines with lowest survival across infectors and days (DGRP_75, DGRP_176, DGRP_177, DGRP_313, DGRP_492) all had an average survival five days-post infection (5 dpi) of lower than 30%, whereas the top five lines (DGRP_38, DGRP_319, DGRP_405, DGRP_822, DGRP_913) had an average survival of at least 94% at 5 dpi (Figure S2b). There was also considerable variance within lines, which can be partially explained by the fact that infections were conducted by multiple people across multiple treatment days. We therefore fitted a model to the data to account for the effects of treatment date and infector identity, and use fitted values from this model in subsequent analyses (Figure 1a).

### 3.2. Identification of Candidate Genes

After applying a *p*-value cut-off of *p* < 10^−7^, we retained 52 genetic variants, most (94.3%) of which were single nucleotide polymorphisms (SNPs), as potentially associated with *E. faecalis* survival in *D. melanogaster* (Figure 1b solid red line, Table S1). This *p*-value threshold results in a fairly high false discovery rate (FDR) of 23.8%. Therefore, to further narrow down the list of candidate genes (GOIs) for validation, we validated only those genes associated with SNPs with *p* < 10^−8^ (which equates to a false discovery rate of 2.4%, Figure 1b dashed red line), with the following exceptions. First, two highly significant (*p* < 10^−9^) SNPs were found in the intron of *krishah* (*kri*), and two additional SNPs with slightly lower significance (*p* < 10^−7^) were found in the 5′ untranslated region (UTR) of *kri* (Figure S3). *kri* is tightly clustered with the genes *S6k* and *mad2* (Figure S3). All four SNPs are also tightly linked making it difficult to infer the causal SNP. SNPs in this region have also been previously identified as being associated with survival after *Pseudomonas aeruginosa* infection in the DGRP [13]. We therefore included all three of these genes as candidates for RNAi knockdown of gene expression. Second, one highly significant SNP (2L_17921046) occurred in a genomic region devoid of genes, and so could not be validated further. Third, we did not test the role of two genes associated with highly significant SNPs (*Ncc69* and *Glc-AT-P*). In total, we identified six candidate genes (hereafter genes of interest, GOI) and four neighbor genes as targets for further analysis via RNAi knockdown (Table S2). For the genomically clustered GOIs *S6k*, *mad2* and *kri*, a single neighbor gene (*CG42272*) was used.

### 3.3. Candidate Gene Analyses

**Figure 2:**
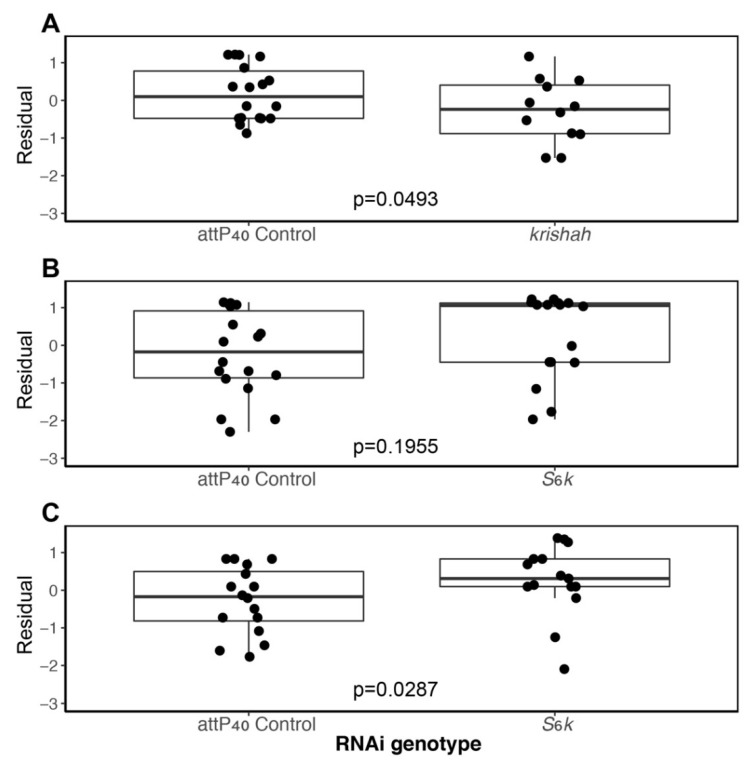
Survival of GOI lines versus empty cassette controls (attP40 control line 36304). (**A**) *krishah* (*kri*, Line 62238) infected with *E. faecalis* at a dose of OD = 1.5; (**B**) *S6 kinase* (*S6k*, Line 42572) infected with *E. faecalis* at a dose of OD = 1.5; (**C**) *S6 kinase* (*S6k*, Line 42572) infected with *E. faecalis* at a dose of OD = 2.4. In all cases, infections were conducted across multiple days. As such, the *y* axis is the residuals from a model controlling for Date effects.

Of the six GOIs, we found significant associations between *E. faecalis* infection and survival with two genes. There was a marginally significant trend for lower survival after infection in *kri*-flies (*n* = 111 GOI and 177 control, Line effect *p* = 0.049, Table 1, Figure 2a). For *S6k*-flies, we noted a non-significant trend towards higher survival in the knockdown lines (*n* = 155 GOI and 172 control, Line effect *p* = 0.196, Table 1, Figure 2b). However, due to high overall survival in this experiment, it was difficult to determine whether knockdown of *S6k* improved survival (Figure 2b). Therefore, we performed a second round of infections with a higher *E. faecalis* dose (OD = 2.4). We indeed found higher survival of *S6k*-flies when infection burden was increased (*n* = 142 GOI and 150 control, Line effect *p* = 0.029, Table 1, Figure 2c). *kri* and *S6k* are clustered on chromosome 3L (Figure S3), suggesting that this region may be associated with the response to *E. faecalis*. However, we did not find any association between infection response and knockdown of two other genes in this region—*mad2* (*n* = 302 GOI and 278 control, Line effect *p* = 0.136, Table 1, Figure S4a) and *CG42272* (*n* = 100 neighbor and 133 control, Line effect *p* = 0.658, Table 1, Figure S4b). Knocking down expression of the other three GOIs, located in different regions of the *D. melanogaster* genome, had no significant effect on survival after *E. faecalis* infected (*p* > 0.05, Table 1, Figure S4 left panel). Likewise, knockdown on neighbor genes had no effect on *E. faecalis* infection survival (*p* > 0.05, Table 1, Figure S4 right panel).

We have previously calculated Tajima’s D for all genes in the DGRP [40]. We ranked all DGRP genes from most negative to most positive and calculated the percentile ranking for each GOI and neighbor gene (Table S2). None of the genes were ranked amongst the highest or lowest 10% of genes in terms of Tajima’s D, although *S6k* had a rather positive Tajima’s D (1.001, 88^th^ percentile), suggesting it may be subject to balancing selection or other forces that promote positive Tajima’s D. For the remainder of the GOIs and neighbor genes, selective forces appear to be similar to the majority of the genes in this population (Table S2).

### 3.4. Role of Bomanin Genes on *E. faecalis* Survival

Bomanins are a newly described group of immune peptides in *Drosophila* [15], and have an important role in response to Gram-positive [15,22,41] and fungal [23] infections. However, to the best of our knowledge, no survival analyses have yet been performed using *E. faecalis* infection of lines where expression of specific Bom genes has been knocked down. We therefore investigated the two genomic regions with clusters of Bom genes more closely. 

**Figure 3:**
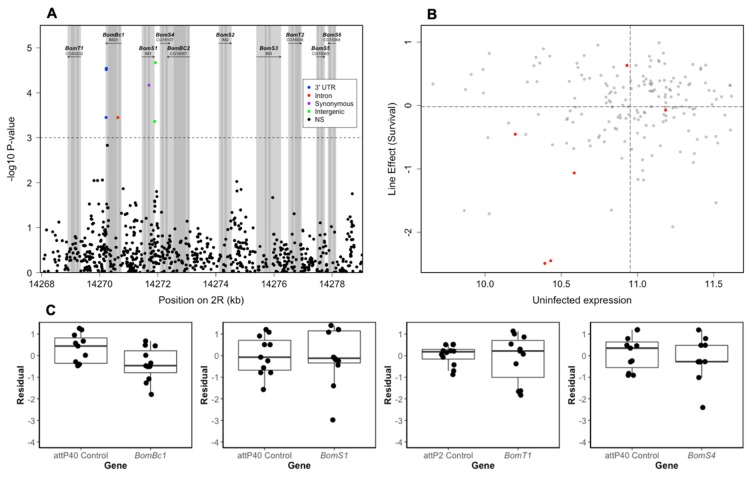
Relationship between Bomanin genes and *E. faecalis* survival. (**A**) Manhattan plot of genetic variants within the Bomanin cluster on chromosome 2R of the *D. melanogaster* genome and their association with *E. faecalis* survival. *p*-value is plotted as −log_10_ *p*-value. Light grey polygons denote the limits of genes, and the dark grey polygons indicate coding sequences within genes. Color of points denotes location of variant with respect to the closest gene, as per legend. (**B**) Expression of the perfectly linked SNPs 2R_14270226 and 2R_14270228 in uninfected DGRP individuals plotted against survival of the same lines after infection with *E. faecalis.* Red dots denote the six lines homozygous for the minor allele. In general, these lines display lowered survival after *E. faecalis* infection (lower left quadrant). Dashed lines indicate means for survival (horizontal line) and uninfected expression (vertical line). (**C**) Survival of Bom-lines versus empty cassette controls after five days of infection with *E. faecalis* at a dose of OD = 1.5. From left to right, *BomBc1, BomS1, BomT1, BomS4*.

The main Bom cluster is located on chromosome 2R and contains 10 of the 12 Bom genes. We noted several SNPs with *p* < 10^−3^ in the 5′ portion of this region, including two perfectly linked SNPs in the 3′ UTR of *BomBc1* with *p* < 10^−5^ (Figure 3a). Because mutations in the UTR can have an effect on gene expression, we next investigated whether baseline (uninduced) *BomBc1* gene expression, data from [25] was influenced by these two linked SNPs, and found that individuals possessing the minor allele tended to have lower expression than those with the major allele (Figure 3b). To investigate the role of Bom genes in response to infection with *E. faecalis,* we knocked down expression of four Bomanin genes (*BomS1, BomBc1, BomT1* and *BomS4*; also known as *IM1, IM23, CG43202* and *CG18107* respectively). We found a significant effect for *BomBc1* such that individuals not expressing this gene had lowered survival compared to controls (*n* = 105 *BomBc1* and 105 control, Line effect *p* = 0.023, Table 2, Figure 3c). Knocking down the other three Bom genes had no significant effect on survival (Table 2). Using Tajima’s D values for the DGRP calculated in Chapman et al. [40], we found that *BomS1* had a Tajima’s D value (1.157) in the 91^st^ percentile of all DGRP genes (Table S3), suggesting it may be subject to balancing selection, as has previously been shown for antimicrobial peptides, including Bomanins [40]. Tajima’s D for the other three Bomanin genes were similar to DGRP genes as a whole (46^th^ to 72^nd^ percentiles, Table S3).

The other Bom cluster, comprising two Bom genes, is located on chromosome 3L. We found no evidence for SNPs associated with immune defense in this region (Figure S5), and so did not investigate these two genes further.

## 4. Discussion

The distribution of survival proportions (Figure 1A) found in the genome wide association study indicates the presence of substantial genetic variation for defense against *Enterococcus faecalis* in *Drosophila melanogaster* even after controlling for the effects of infection date, infector identity and *Wolbachia* status. Since *E. faecalis* is present in wild populations of *D. melanogaster* [14], this genetic variance may have arisen due to selective pressure exerted in nature [42]. Resistance, whereby the host works to clear an infection; and tolerance, whereby the host works to limit the damage caused by infection; can both be evolutionary stable strategies for hosts, and selection will promote whichever strategy (or combination of strategies) optimizes fitness [9,43]. However, because we simply measured survival after infection, we cannot determine whether genetic variation arose via selection on tolerance or resistance to *E. faecalis* in the DGRP. Furthermore, the extent of the selection pressure exerted specifically by *E. faecalis* as opposed to other bacterial pathogens that *Drosophila* encounter in the wild is an open question, which will be influenced by numerous factors including pathogen encounter rates, pathogen infectivity and pathogen virulence. It may be that selection acts largely at the level of pathogen type (e.g., viral, bacterial, fungal, macro-parasitic), or at the level of classes within these classifications (e.g., Gram-positive vs Gram-negative bacteria, DNA vs RNA viruses) [42,44]. This could explain why one of the main classes of immune response genes in *Drosophila*, the antimicrobial peptides, are thought to have rather broad-spectrum activities [45,46] rather than targeting specific pathogens [22,47]. It has recently been shown that infection with *E. faecalis* elicits lower AMP induction than other pathogens in *Drosophila* when infected at low dose [31]. Beyond broad-spectrum protective immune genes however, it may be that other genes have evolved to respond to specific pathogen threats. 

The genetic variants we identified via GWAS as significantly associated with *E. faecalis* survival were SNPs associated with six genes, none of which were associated with canonical immune genes or GO terms related to immunity. *Islet antigen 2* (*IA-2*) is part of the protein tyrosine phosphatase superfamily [48], and has been found to be involved in gut development during metamorphosis via modulation of insulin and hexokinase expression [49]. The 3′ end of the IA-2 protein contains a conserved *PTP_DSP_cys* protein domain characterized by a CxxxxxR catalytic loop. The PTP superfamily of proteins is part of the tyrosine phosphorylation/dephosphorylation regulatory mechanism and help cells respond to physiological changes in their environment [50]. *CG30377* has been implicated in copper ion homeostasis [51]. It contains no known conserved protein domains. *CG6767* is a kinase involved in purine and pyrimidine base metabolism [52], is required for glial cell differentiation [53] and affects olfactory behavior [54]. The CG6767 protein is largely comprised of a conserved *PrsA* domain. This protein superfamily is involved in nucleotide and amino acid transport and metabolism [50]. *mad2* is a mitotic spindle assembly checkpoint protein, expressed in mitotic cells and important for normal cell division processes [55,56,57]. The protein contains a *HORMA* protein domain. This protein superfamily is thought to be involved in recognizing aberrant chromatin states that arise due to DNA double stranded breaks, DNA adducts and non-attachment to the mitotic spindle [50]. *krishah* (*kri*) encodes an enzyme that regulates larval growth, pre-pupal and pupal viability, and adult longevity and is homologous to human uracil phosphoribosyltransferase [58]. The Kri protein contains a *UPRTase* domain which belongs to the *PRTases_typeI* superfamily of protein domains. Type I PRTases catalyze the displacement of α-1′-pyrophosphate of 5-phosphoribosyl-alpha1-pyrpphosphate (PRPP) by a nitrogen-containing nucleophile [50]. *S6 kinase* (*S6k*) is a ribosomal protein kinase [59] that acts as an effector of the TORC1 growth-regulatory complex [60] and references therein] and appears to regulate various brain activities, such as synapse development [61], behavioral responses to hunger [62] and ethanol induced sedation [63].The S6k protein is largely comprised of the conserved protein domain *STKc_p70s6K* which belongs to the *PKc_like* superfamily of catalytic domains which catalyze transfer of a γ-phosphoryl group from ATP to hydroxyl groups in serine, threonine, or tyrosine residues of proteins [50]. We used the STRING database [64] to investigate known protein interactions for each of these six genes. Several genes in the *S6k* network are involved in Target of Rapamycin (TOR) signaling (Figure S6). Rapamycin can act as an immunosuppressant in *Drosophila*, and has antifungal properties [65]. Downregulation of the TOR pathway has been shown to improve immune defense in female *D. melanogaster* [66]. We did not identify any of our other candidate genes as belonging to protein networks involving known immunity genes (Figure S6).

However, while none of the six candidate genes have previously been identified as having canonical immune functions, there is some evidence that a subset of them may have immune-related roles. First, *CG30377* is somewhat induced (up to 3-fold) by *Escherichia coli* in S2 cells [67]. Second, a separate GWAS identified two SNPs associated with the clustered genes *kri*, *S6k* and *mad2* as significantly associated with *Pseudomonas aeruginosa* defense in the DGRP [13]. Both SNPs were synonymous substitutions in *kri* and therefore occurred upstream of *S6k* and downstream of *mad2*. Interestingly, both *E. coli* and *P. aeruginosa* are Gram-negative bacteria, whereas *E. faecalis* is Gram-positive. In *Drosophila,* bacterial pathogens are controlled by two different innate immune pathways. The immune deficiency (IMD) pathway is generally triggered by Gram-negative bacteria, whereas the Toll pathway is generally triggered by Gram-positive bacteria and fungi [68,69]. The fact that our GWAS with a Gram-positive bacterium identified a subset of genes that had previously been identified as responding to Gram-negative bacteria suggests these genes may be stimulated upstream of the Toll and IMD pathways or through cross-talk between these pathways and others. In particular, IMD activation downregulates the TOR pathway [70], and TOR suppression improves immunity in *Drosophila* [66]. Given the role of *S6k* as an effector of TOR signaling (Figure S6), this may explain why this gene has been previously identified as playing a role in defense against Gram-negative infections. In mammals, mTOR has complex interactions with Toll-like receptor innate immune responses [71], which may help explain the role of *S6k* in mediating survival against a Gram-positive bacterium in *Drosophila.* Consistent with this, we found that reducing the expression of *S6k* improves survival against *E. faecalis*, suggesting this may be mediated via downregulation of the TOR pathway or an intermediary.

Validation by RNAi knockdown confirmed only two of the six candidate genes as having a significant association with *E. faecalis* survival when knocked down. *S6k* appears to be a negative regulator of *D. melanogaster* defense against *E. faecalis* infection, such that knocking down expression of this gene improves survival prospects for the fly. In contrast, *krishah* appears to be a positive regulator of the *D. melanogaster* defense against *E. faecalis* infection, such that knocking down expression of this gene reduces fly survival. 

It is important to note that our GWAS was conducted with a single set of conditions (i.e., males infected with a specific *E. faecalis* dose and maintained on cornmeal-molasses-yeast food at 22 °C on a 12-h day/night cycle). A different set of conditions may have yielded different significant GWAS hits. For example, we have previously shown that dietary composition affects immune defense [12]. Furthermore, other factors, such as genome size, could influence identification of significant GWAS hits. Genome size can affect phenotypic traits such as survival to adulthood in the DGRP, apparently via gene expression changes that affect metabolism [72]. It is noteworthy that lines with large genome sizes upregulate genes involved in TOR signaling [72], which could have implications for immunity, as discussed above.

Bomanins are a group of AMP-like genes that appear to be key effectors of the *Drosophila* immune response to Gram-positive infections [15,22,23]. As such, we anticipated that Bomanin genes may be involved in response to *E. faecalis* infection in the DGRP. While we found some evidence for associated SNPs in the Bom region (Figure 3A), and that uninfected expression of the two (perfectly linked) SNPs in the 3′UTR of *BomBc1* were potentially associated with defense amongst DGRP lines (Figure 3B), our RNAi candidate gene results were equivocal. *BomBc1*-mutants had significantly lower survival. However, we found no associations with the other three genes we tested (Figure 3C). It is possible that Bomanins have some functional redundancy, such that no one Bomanin protein is essential for controlling a specific infection, instead a cocktail of Bom proteins may help to bring the infection under control. Indeed, Clemmons et al. [15], Hanson et al. [22] and Lin et al. [41] have all found that mutant flies (Bom^Δ55C^) lacking ten of the twelve Bom genes have extremely low survival when infected with *E. faecalis*, whereas our knockdown of individual Bom genes generally had little effect on survival, with the exception of *BomBc1*. Synergistic effects of AMPs have previously been shown in-vitro, e.g., [73,74,75] and in-vivo e.g., [76]. Additionally, it has recently been proposed that short-form Bomanins are particularly important for the *Drosophila* response to *Candida* infection, whereas bicipital Bomanins (e.g., *BomBc1*) may respond to other pathogens such as Gram-positive bacteria [41].

This work, dissecting the relationship between genetic variation and *E. faecalis* survival in the DGRP, improves our understanding of immunity related phenotype-genotype interactions in *Drosophila.* Such knowledge also elucidates how host-pathogen interactions shape the evolutionary trajectory of host populations.

## Figures and Tables

**Table 1:** Results of generalized linear models with a binomial distribution and a logit link fitted to survival data after *E. faecalis* (EF) infection. Genes of Interest (GOI) are listed above their genomic neighbor comparison. DF is degrees of freedom. *p* values less than 0.05 are shown in bold.

Gene	Type	Factor	Deviance	Df	*p*-Value
*Krishah*	GOI	Line	3.8655	1	**0.0493**
		Date	14.7613	3	**0.0020**
*S6k* ^1^	GOI	Line	1.6755	1	0.1955
		Date	7.0270	4	0.1345
*S6k* ^2^	GOI	Line	4.7836	1	**0.0287**
		Date	1.0198	2	0.6006
*mad2*	GOI	Line	2.2275	1	0.1356
		Date	5.9814	1	**0.0145**
*CG42272*	neighbor	Line	0.5070	1	0.4765
		Date	6.3657	2	**0.0415**
*CG6767*	GOI	Line	0.3116	1	0.5767
		Date	6.0510	3	0.1092
*CG6761*	neighbor	Line	3.1126	1	0.0777
		Date	3.7781	3	0.2864
*IA2*	GOI	Line	1.5029	1	0.2202
		Date	1.9210	3	0.5890
*Star*	neighbor	Line	3.0205	1	0.0822
		Date	0.8459	2	0.6551
*CG30377*	GOI	Line	0.1081	1	0.7423
		Date	0.0669	1	0.7959
*Dgk*	neighbor	Line	0.0193	1	0.8894
		Date	2.9014	3	0.4071

^1^ Infections with *E. faecalis* at a dose of OD = 1.5, ^2^ Infections with *E. faecalis* at a dose of OD = 2.4.

**Table 2:** Results of generalized linear models with a binomial distribution and a logit link fitted to survival data for Bom-lines after *E. faecalis* (EF) infection. Df is degrees of freedom. *p*-values less than 0.05 are shown in bold.

Gene	Factor	Deviance	Df	*p*-Value
*BomBc1*	Line	5.1995	1	**0.0226**
	Date	3.3653	2	0.1859
*BomS1*	Line	0.2420	1	0.6228
	Date	9.8015	2	**0.0074**
*BomT1*	Line	0.0041	1	0.9491
	Date	10.8707	2	**0.0044**
*BomS4*	Line	0.7496	1	0.3866
	Date	2.2465	2	0.3252

## Supplementary Materials

The following are available online at https://www.mdpi.com/2073-4425/11/2/234/s1, Figure S1: GWAS analysis data, Figure S2: Manhattan plot of genetic variants in an 18 kb window on chromosome 3L, Figure S3: Survival of GOIs (left panel) or neighbor genes (right panel) compared to empty cassette controls, Figure S4: Manhattan plot of genetic variants within the Bomanin cluster on chromosome 3L, Table S1: List of genetic variants with significant GWAS hits (*p* < 10^−7^), Table S2: List of candidate genes and controls and Table S3: List of Bomanin lines used in this study.
