# Supplementary material for: The Genetic Basis of Natural Variation in Drosophila melanogaster Immune Defense against Enterococcus faecalis
Source: Genes (Basel). 2020 Feb 22;11(2):234. doi: 10.3390/genes11020234 (PMC7074548; doi:10.3390/genes11020234)
Supplement: Supplementary file 1 [file genes-11-00234-s001.zip › SuppFigs.docx]

**Supplementary Figures**


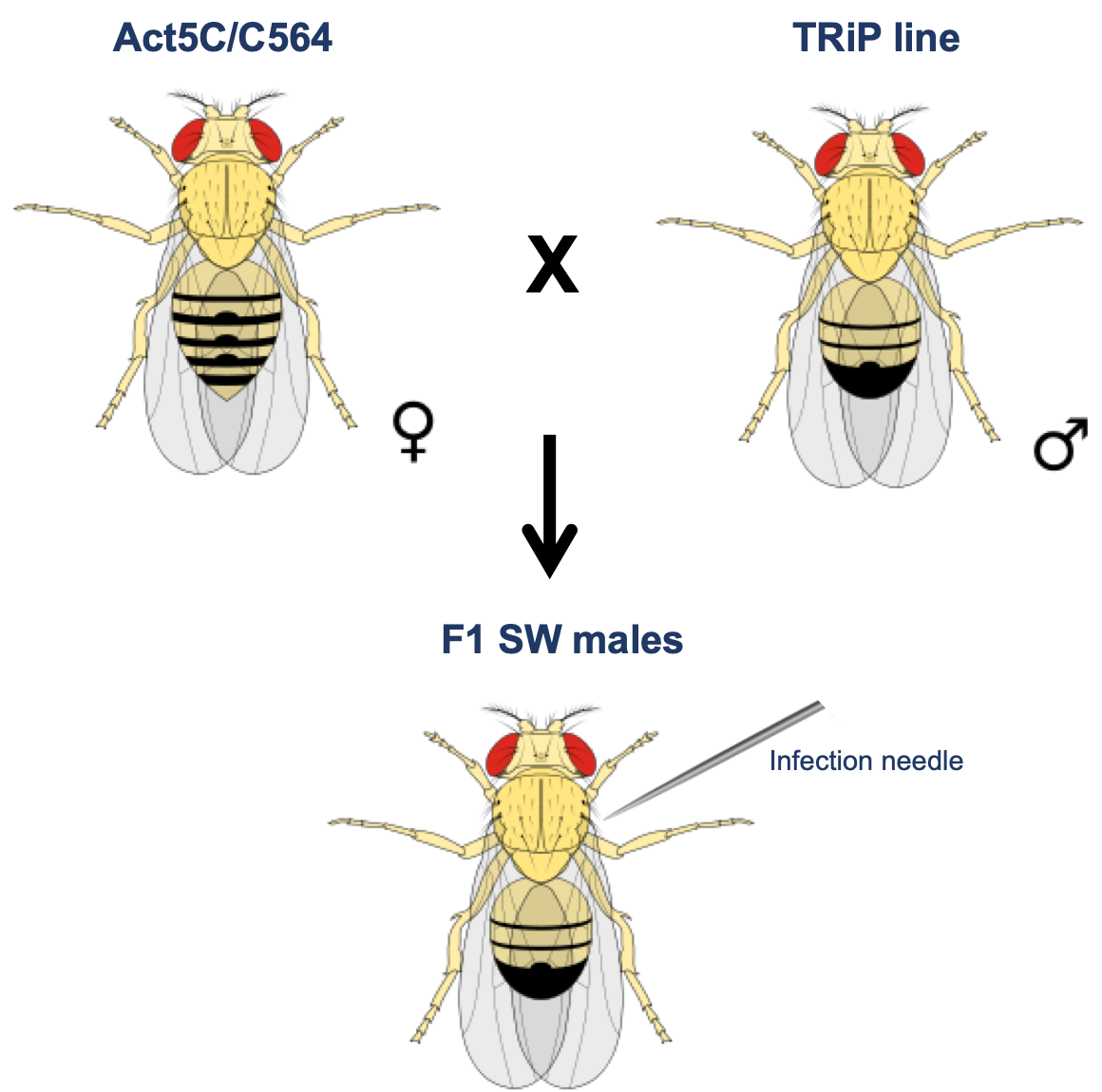


**Figure S1.** Schematic of crossing regime for infection of RNAi knockdown lines. A virgin Act5C-GAL4 or C564-GAL4 female was crossed to a male from the relevant TRiP line (see M&M of main paper). F1 straight winged (SW) males were collected onto new food and infected with a sterile needle when 4-7 days old. Fly images used under a Creative Commons CC0 1.0 Universal Public Domain Dedication license.


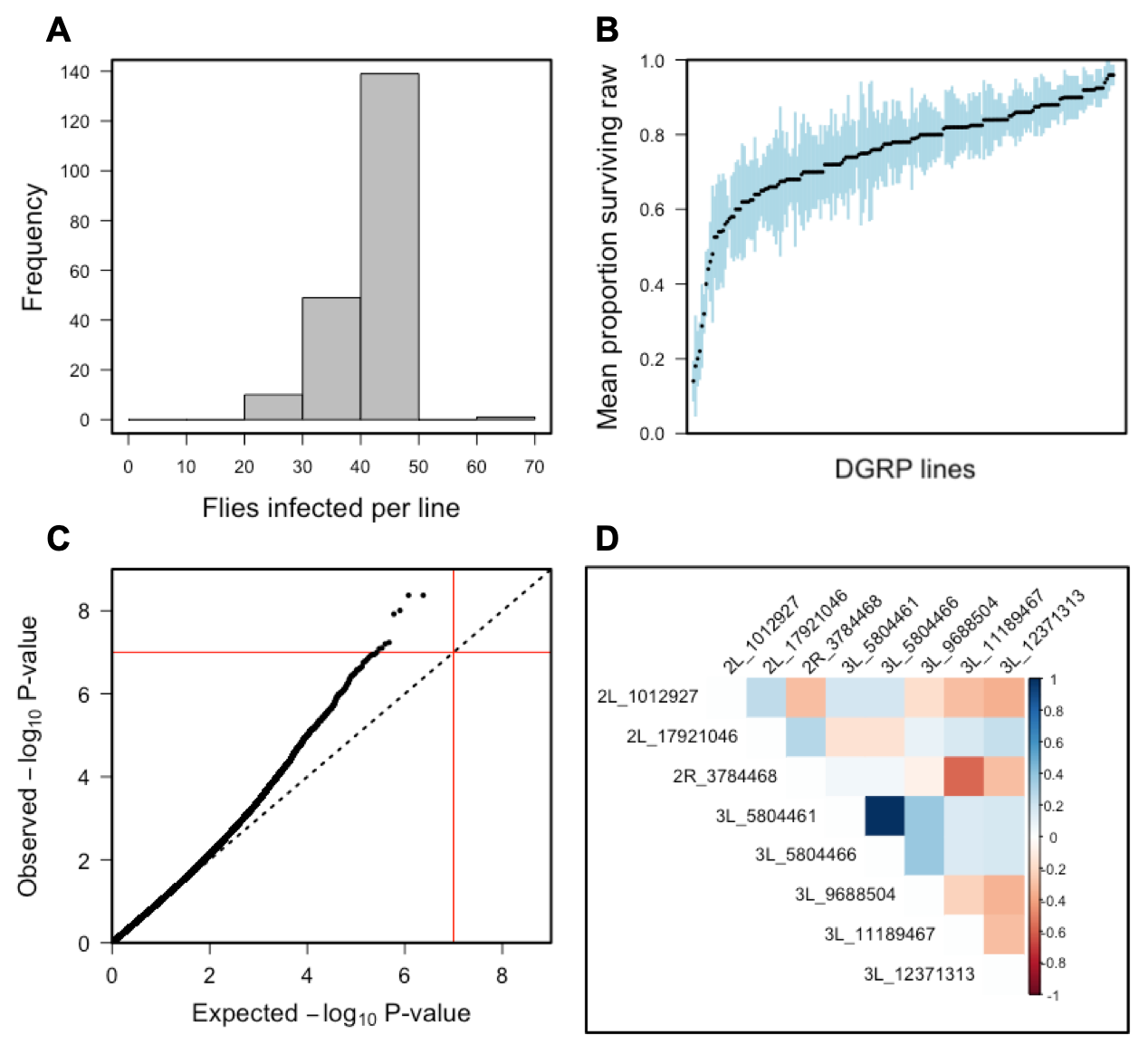


**Figure S2.** GWAS analysis data. A) Number of flies infected per line; B) Distribution of survival rates amongst DGRP lines infected with *E. faecalis***.** Black line represents fitted average for a model of survival taking into account the fixed effects of infector and date of infection. The plot is sorted by increasing survival, and blue bars indicate standard error of the mean. C) QQ plot of expected versus observed log_10_ *P*-values. Deviation from a 1:1 line is a common observation for GWAS analyses using the DGRP. D) Correlation between 9 variants (all of which are SNPs) with *p* < 10^-8^ and associated with genes selected for GOI analysis. Red shading indicates negative correlation and blue shading indicates positive correlation; darker shades indicate higher levels of linkage disequilibrium.

**
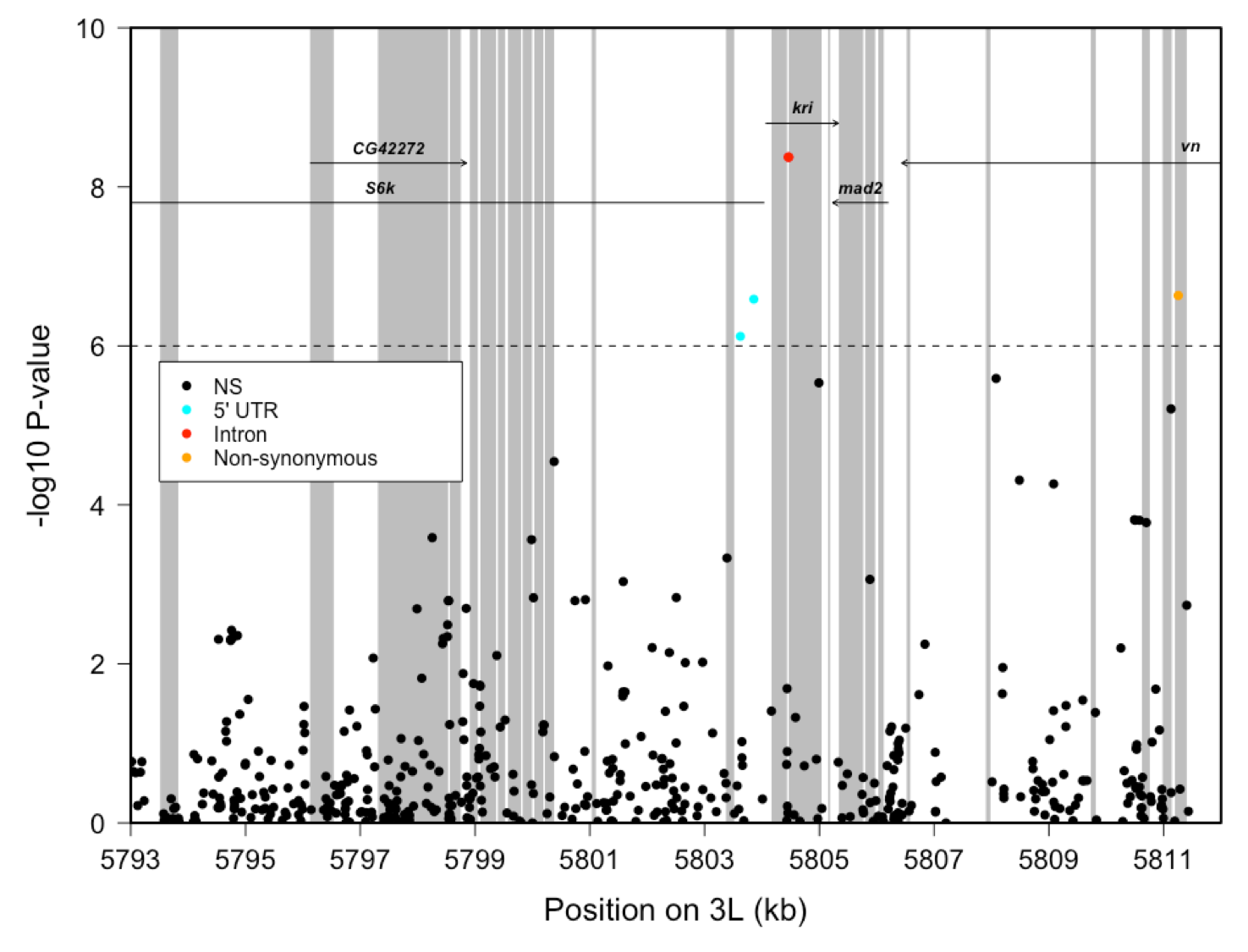
**

**Figure S3.** Manhattan plot of genetic variants in an 18 kb window on chromosome 3L containing the genes *S6k, CG42272, kri, mad2* and *vn*. Because these genes overlap to various extents (e.g. *CG42272* is wholly overlapped by *S6k*, and the UTRs of *kri* and *mad2* overlap), shading denotes coding sequences within genes, rather than the entire gene span.

**
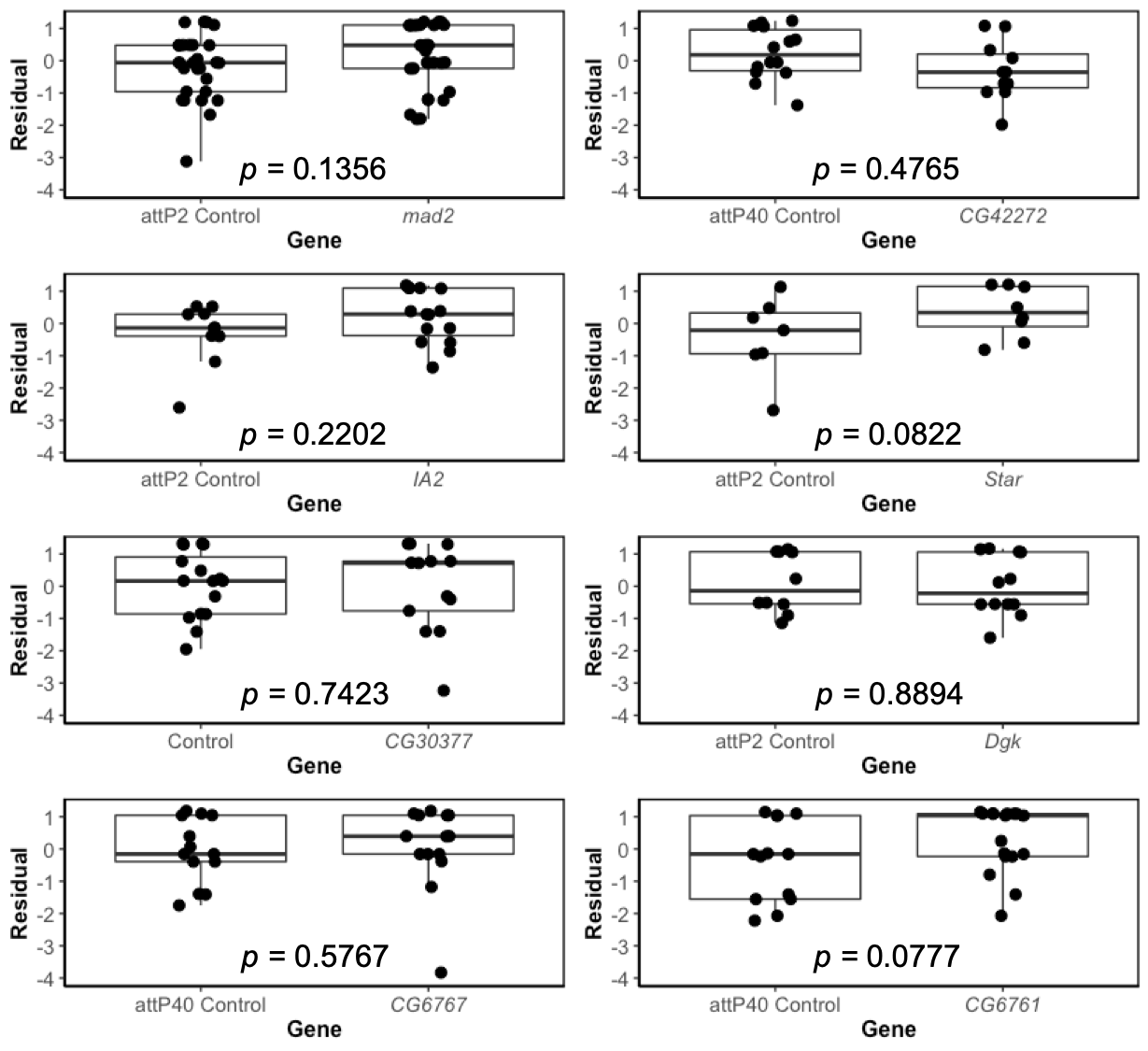
**

**Figure S4.** Survival of GOIs (left panel) or neighbour genes (right panel) compared to empty cassette controls (36304 or 306303). A) *mad2* (Line 44430); B) *CG42272* (Line 63602); C) *CG6767* (Line 60088); D) *CG6761* (Line 63638); E) *IA-2* (Line 33672); F) *Star* (Line 38914); G) *CG30377* (Line 51386); H) *Diacyl glycerol kinase* (*Dgk*, Line 36745). In all cases, infections were conducted by a single person (JRC or MAD) across multiple days. As such, the y axis is the residuals from a model controlling for Date effects.


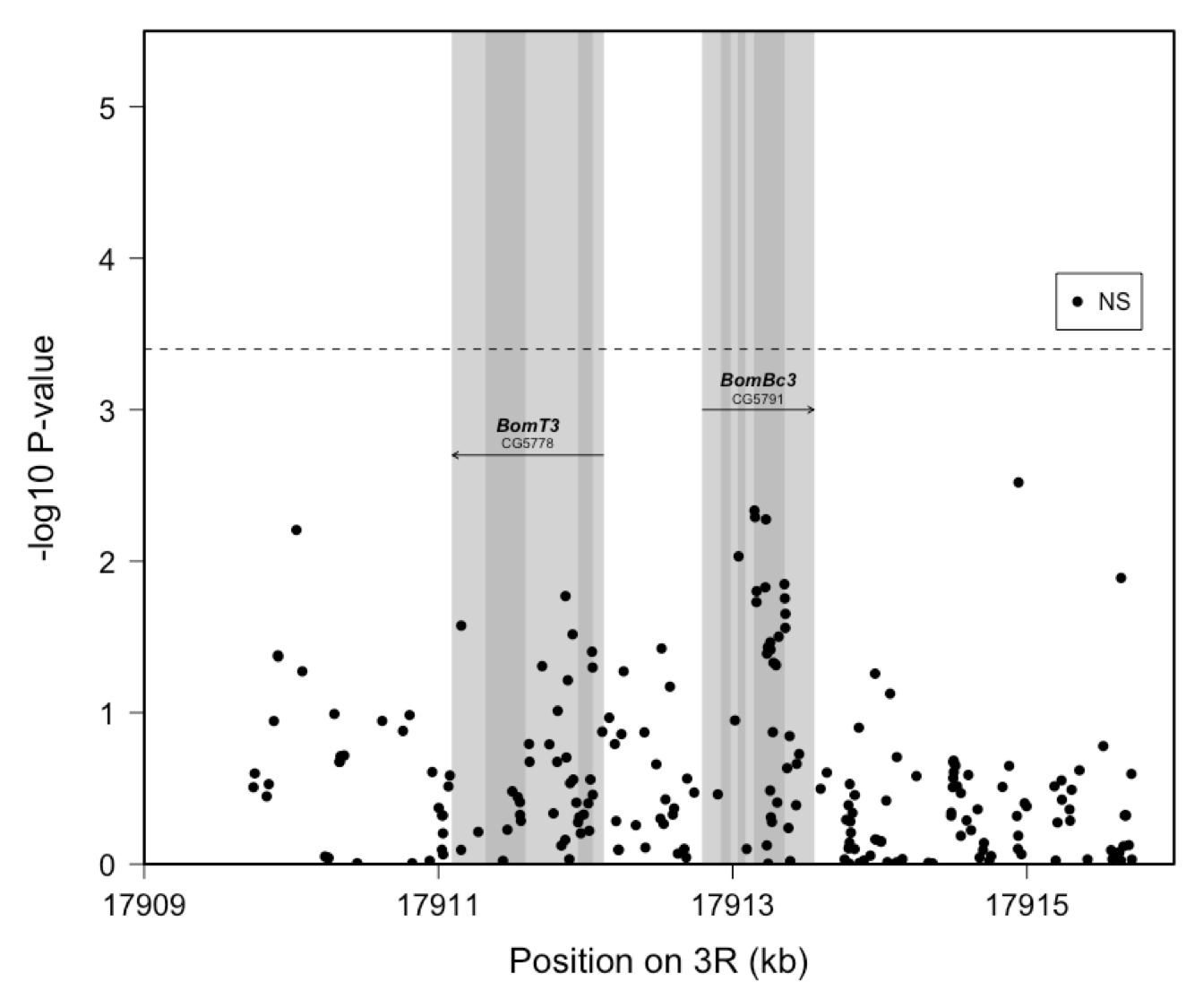


**Figure S5.** Manhattan plot of genetic variants within the Bomanin cluster on chromosome 3L of the *D. melanogaster* genome and their association with *E. faecalis* survival. *P*-value is plotted as -log_10_ *P-*value. Light grey polygons denote the limits of genes, and the dark grey polygons indicate coding sequences within genes.

**
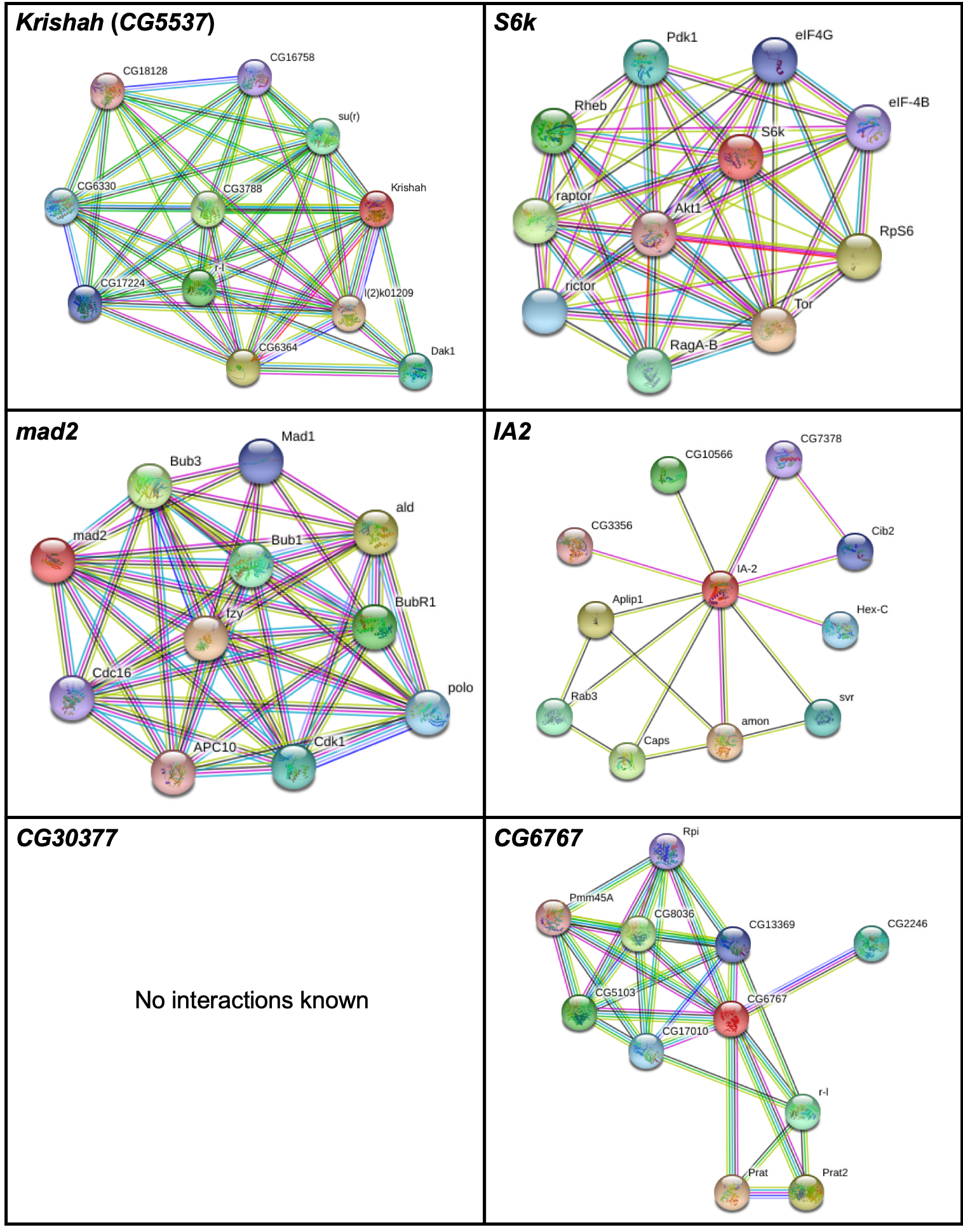
**

**Figure S6.** Protein interaction networks predicted by STRING (<https://string-db.org>) for the six genes with significant GWAS hits. In all cases, light blue and pink lines are known interactions from curated databases and experimentally determined, respectively; dark green, red and navy blue lines are predicted interactions, specifically gene neighbourhood, gene fusion and gene co-occurrence respectively; lime green, black and mauve lines are other protein interactions, predicted via textmining, co-expression, and protein homology respectively. Filled nodes are proteins with tertiary structure information while empty nodes are proteins without tertiary structure information.
